# Declaration of Santo Domingo: commitment to improve education, regulations, public policies, and access to peritoneal dialysis in Latin America

**DOI:** 10.1590/2175-8239-JBN-2026-SA01en

**Published:** 2026-04-03

**Authors:** Gustavo L. Moretta-Pedernera, Alfonso Ramos-Sánchez, Laura Cortés-Sanabria, Rosana Chaúd-Covarrubias, Guillermo Álvarez-Estévez, Mauricio Uribe, Vicente Sánchez-Polo, José Carolino Divino-Filho, Natalia M. Wasmuth-Troche, Ledisbeth Batista-Buitrago, Marta Avellán-Boza, Rodrigo Orozco-Bass, Héctor Martínez-Eusebio, René Soto-Velásquez, María Marina Papaginovic-Leiva, Martín Gómez-Luján, Sandra Rodríguez-Manzano, José Manuel Rodríguez-Chagolla, Daniel Alejandro Molina-Comboni, Yanet Álvarez-González, Pedro Javier Dávila Crespo, Erwin Iván Campos, José A. Moura-Neto, Magda Hinojosa, Lourdes Carolina Vásquez-Jiménez, Gloria G. García-Villalobos, Gabriela González, Eliana Dina-Batle, José R. Álvarez-Novoa

**Affiliations:** 1 Capítulo Latinoamericano de Diálisis Domiciliaria (LACDD), Latin America.; 2 Universidad de San Andrés, Buenos Aires, Argentina.; 3 Silher Corporación, Lima, Peru.; 4 Macrotech Farmacéutica, Mexico City, Mexico.; 5 Sociedad Latinoamericana de Nefrología e Hipertensión (SLANH), Latin America.; 6 Universidad de Guadalajara, Centro Universitario de Tonalá, Jalisco, Mexico.; 7 Fondo Intangible Solidario de Salud, Ministerio de Salud, Lima, Peru.; 8 CEDIMAT, Santo Domingo, Dominican Republic.; 9 Asociación Colombiana de Nefrología e Hipertensión, Bogotá, Colombia.; 10 Universidad Pontificia Bolivariana, Medellín, Colombia.; 11 Universidad de San Carlos, Ciudad de Guatemala, Guatemala.; 12 Karolinska Institutet, Stockholm, Sweden.; 13 Sociedad Paraguaya de Nefrología, Asunción, Paraguay.; 14 Hospital Central del Instituto de Previsión Social, Asunción, Paraguay.; 15 Sociedad Panameña de Nefrología e Hipertensión, Panama City, Panama.; 16 Hospital Regional Rafael Hernández, Chiriquí, Panama.; 17 Asociación Costarricense de Nefrología, San José, Costa Rica.; 18 Universidad de Costa Rica, San José, Costa Rica.; 19 Hospital Enrique Baltodano, Liberia, Guanacaste, Costa Rica.; 20 Sociedad Chilena de Nefrología, Santiago, Chile.; 21 Universidad de los Andes, Santiago, Chile.; 22 Sociedad Dominicana de Nefrología, Santo Domingo, Dominican Republic.; 23 Hospital Dr. Francisco Moscoso Puello, Santo Domingo, Dominican Republic.; 24 Asociación de Nefrología e Hipertensión Arterial El Salvador, San Salvador, El Salvador.; 25 Instituto Salvadoreño del Seguro Social, San Salvador, El Salvador.; 26 Centro de Hemodiálisis de El Salvador, San Salvador, El Salvador.; 27 Sociedad Argentina de Nefrología, Buenos Aires, Argentina.; 28 Universidad Católica Argentina, Buenos Aires, Argentina.; 29 Complejo Médico Churruca-Visca, Buenos Aires, Argentina.; 30 Sociedad Peruana de Nefrología, Lima, Peru.; 31 Universidad Nacional Federico Villarreal, Lima, Peru.; 32 Hospital Regional Antonio Musa, San Pedro de Macorís, Dominican Republic.; 33 Colegio de Nefrólogos de México A.C., Mexico City, Mexico.; 34 Universidad Nacional Autónoma de México, Mexico City, Mexico.; 35 Centro Médico ISSEMYM, Toluca, Mexico.; 36 Sociedad Boliviana de Nefrología, La Paz, Bolivia.; 37 Hospital Guaracachi, Santa Cruz, Bolivia.; 38 Caja Petrolera de Salud, Santa Cruz, Bolivia.; 39 Clínica Incor, Santa Cruz, Bolivia.; 40 Sociedad Cubana de Nefrología, Havana, Cuba.; 41 Instituto de Nefrología, Havana, Cuba.; 42 Asociación Guatemalteca de Nefrología, Guatemala City, Guatemala.; 43 Unidad Nacional de Atención al Enfermo Renal Crónico, Guatemala City, Guatemala.; 44 Pontificia Universidad Católica Madre y Maestra, Santo Domingo, Dominican Republic.; 45 Hospital Salvador Bienvenido Gautier, Santo Domingo, Dominican Republic.; 46 Sociedade Brasileira de Nefrologia, São Paulo, Brazil.; 47 Escola Bahiana de Medicina e Saúde Pública, Salvador, Brazil.; 48 Comité Andino de Salud Renal, Andean Region.; 49 Organismo Andino de Salud – Convenio Hipólito Unanue (ORAS-CONHU), Lima, Peru.; 50 Universidad Nacional de Asunción, Asunción, Paraguay.; 51 Instituto Mexicano de Investigaciones Nefrológicas, Mexico City, Mexico.; 52 Hospital General de México Dr. Eduardo Liceaga, Mexico City, Mexico.; 53 Universidad de Buenos Aires, Buenos Aires, Argentina.; 54 Hospital de Clínicas José de San Martín, Buenos Aires, Argentina.; 55 Hospital Metropolitano de Santiago, Santiago, Dominican Republic.; 56 Sociedad Nicaragüense de Nefrología, Managua, Nicaragua.; 57 Universidad Nacional Autónoma de Nicaragua, Managua, Nicaragua.; 58 Hospital Bautista, Managua, Nicaragua.; 59 Hospital Escuela Lenin Fonseca Martínez, Managua, Nicaragua.

**Keywords:** Peritoneal Dialysis, Santo Domingo Declaration, Education, Medical, Harmonization, International Standards, SONG-PD, ESENeph, Public Policy, Equitable Access, Renal Health

## Abstract

The Santo Domingo Declaration highlights the challenges and opportunities to improve education, regulations, public policies, and access to peritoneal dialysis (PD) in Latin America. It emphasizes the need to address structural inequalities in training and care, establishing minimum competency standards and strengthening health infrastructure. Inspired by initiatives such as the Standardised Outcomes in Nephrology–Peritoneal Dialysis (SONG-PD) and the European Specialty Examination in Nephrology (ESENeph), the declaration underlines the importance of harmonizing nephrologist training to promote standardised and internationally aligned competencies. Additionally, it outlines strategies to prioritize patients’ quality of life, ensure accessibility and economic sustainability, and foster the implementation of satellite units and innovative technologies. Finally, it calls for intersectoral collaboration to ensure inclusive and equitable healthcare in the region.

## Introduction

The Latin American Workshop on Peritoneal Dialysis Access, organised by the Latin American Society of Nephrology and Hypertension (SLANH) and the Latin American Chapter of Home Dialysis (LACDD), brought together a group of nephrology experts, representatives of scientific societies, and health authorities in Santo Domingo, Dominican Republic. The objective was to discuss the challenges facing the region concerning access, regulations, public policies, and education in peritoneal dialysis (PD).

## Regional Context of Chronic Kidney Disease

Chronic kidney disease is a silent pandemic in Latin America, driven by the continuous increase in conditions such as diabetes mellitus and hypertension, which raise the demand for renal replacement therapies, including PD. Despite its recognised cost-effectiveness and favourable clinical outcomes, only 12% of patients in the region have access to this treatment modality.

According to the World Health Organization, universal health coverage means that all people can access quality health services without facing financial hardship, especially those belonging to the most vulnerable groups. However, in Latin America, disparities in access to healthcare, based on socioeconomic, demographic, and ethnic factors, contribute to significant differences in life expectancy and quality of care^1^. The lack of coverage generates health inequities, which can be corrected through appropriate policies^2^.

In the region, nearly a third of people report not seeking care when they need it. Financial barriers, lack of resources (such as health personnel and medicines), and geographical difficulties exacerbate the situation. These barriers are particularly severe in the poorest quintiles of the population, demonstrating structural inequity in access to medical care, including PD3.

## Challenges and Opportunities for Peritoneal Dialysis

Regulations in Latin America are heterogeneous regarding the organisation and administration of peritoneal dialysis centres, which have a direct impact on both the quality of treatment offered and the expansion of this therapy in the region^4^. Furthermore, nephrology specialisation programmes show marked heterogeneity in their duration and workload, ranging from 2 to 5 years, as well as in their curricular structures, thus hindering the standardisation of training^5^.

International experiences have demonstrated the successful implementation of PD as a first therapeutic option, known as the “PD First” policy. In countries such as Thailand and Hong Kong^6^, this strategy has been observed to significantly improve coverage for patients with chronic kidney disease. In Hong Kong, for example, the “PD First” policy has led to an increase in patient survival, along with a reduction in costs compared with haemodialysis. In Thailand, the implementation of this policy facilitated more equitable access to renal therapy in both rural and urban areas, contributing to the expansion of treatment nationwide and helping to reduce haemodialysis waiting lists.

## Standardisation of Peritoneal Dialysis Outcomes: Global Initiatives and the Latin American Context

Globally, initiatives such as the Standardised Outcomes in Nephrology–Peritoneal Dialysis (SONG-PD)^7^ have demonstrated the importance of establishing a standardised set of key outcomes for clinical practice in PD. This project involves a wide range of stakeholders, including patients, carers, clinicians, and other interested parties, who participate in identifying shared priorities. This ensures that reported outcomes are meaningful and relevant for clinical and policy decision-making.

However, in Latin America, before being able to advance towards outcome standardisation, as proposed by SONG-PD, it is essential that countries in the region address basic structural challenges. It is necessary to reorganise and strengthen crucial processes, which include improving the training of medical professionals and patients so that they are adequately prepared to use PD. Furthermore, it is crucial to implement robust and clear public policies, along with regulatory frameworks that support both the expansion of and equitable access to this treatment modality.

A key aspect is to clearly define the competencies that graduates of nephrology specialisation programmes should develop, as many programmes do not do so explicitly, which limits the clarity of the professional profile.

Regarding patient education, the use of assessment tools, such as rubrics, can be key to ensuring that patients acquire the necessary competencies to manage their treatment at home. These tools allow for an objective evaluation of patient progress in areas such as biosafety, management of connectology, and management of complications, which are crucial aspects for improving clinical outcomes^8^. Outcome standardisation, while a mid- to long-term goal, cannot be effectively implemented without an adequate infrastructure to support these efforts. This requires a comprehensive approach that addresses not only the lack of specialised human resources but also inequalities in access to renal therapies, problems that persist in much of Latin America.

The experience of countries such as Thailand and Hong Kong demonstrates that both coverage and clinical outcomes can be improved if structural policies prioritising PD as the first option are implemented^9^.

This declaration seeks to establish a regional consensus to improve education, regulations, public policies, and access to PD, ensuring that these foundations are strengthened before moving towards global standards such as those suggested by SONG-PD.

During this workshop, the main barriers were analysed, and strategies were proposed to improve regulations, public policies, access, and education in PD in Latin America. The signatories of this declaration represent a consensus on the urgent need to implement concrete actions based on these pillars.

## Consensus Methodology (Delphi Method)^10^

To reach a consensus on improving education, regulations, public policies, and access to PD in Latin America, a methodological approach similar to the Delphi method was used, adapted to the needs and characteristics of the region. This approach allowed for structured, iterative, and collaborative participation by nephrology experts and representatives of various scientific societies. The key elements of the process are described below.

### Initial Surveys of Nephrologists and Scientific Society Representatives

The process began with data collection through two surveys:

– Survey of nephrologists: directed at 362 nephrologists in Latin America with the aim of evaluating the current state of PD education, identifying training gaps, and assessing the implementation of modern pedagogical approaches, such as andragogy, as well as evaluating clinical practice. Table 1 presents a classification of the main domains evaluated in the PD survey, along with examples of questions for each domain and the type of question used. There are two different types of questions: closed questions, which aimed to elicit concrete and specific answers, and Likert-type questions, which allow respondents to self-assess their degree of confidence or preparedness in different aspects of their training. This combination of formats provides both objective data and a more qualitative appreciation of the educational experience in PD.
– Survey of scientific society representatives: 27 representatives were surveyed to collect data on education, regulations, policies, and access to PD. Tables 2 to 4 present the main domains evaluated. Through closed, Likert-type, and open questions, opinions were gathered on regulations, compliance with public policies, funding, and barriers faced by PD programmes. This analysis provides a comprehensive view of the areas requiring action and improvement to ensure better access to PD in Latin America. Tables 2 to 4 also present examples of the questions asked and the type of question used in each domain.

**Table 1:** Domains and example questions from the survey of Latin American nephrologists

Domain	Example question	Question type
Demographics and Training	In what year did you obtain your specialist degree in nephrology?	Closed
PD Experience	How many hours did you dedicate to PD vs. haemodialysis during your training?	Closed
Procedures and Technical Skills	Did you perform peritoneal catheter placement during your training?	Closed
Participation in Management and Evaluation Activities	Did you participate in clinical review meetings with the multidisciplinary team?	Closed
Complementary Training	Have you taken courses in PD outside of your postgraduate programme?	Closed
PD Experience	How competent do you feel in prescribing CAPD/APD?	Likert
Procedures and Technical Skills	How competent do you feel in managing infectious complications?	Likert
Participation in Management and Evaluation Activities	How competent do you feel in interacting with the multidisciplinary team?	Likert

Abbreviations – PD: Peritoneal Dialysis; APD: Automated Peritoneal Dialysis; CAPD: Continuous Ambulatory Peritoneal Dialysis.

**Table 2:** Domains and example questions from the survey of Latin American experts on legislation and public policies

Domain	Example question	Question type
PD regulations	Do you believe that PD is the best option for initiating dialysis in incident patients?	Likert (1–5)
Public policies for PD prioritisation	Does your country have regulations for the development of PD programmes?	Closed (yes/no/maybe)
Accessibility and financing of PD	In your country, does everyone have the right to PD financing?	Closed (yes/no/maybe)
Proposal for a regulatory framework	Do you believe that the existence of regulations would favour the development of PD as the first dialysis option?	Closed (yes/no)
Barriers and actions for PD implementation	What do you believe are the two main barriers to implementing PD programmes in your country?	Open-ended

Abbreviation – PD: Peritoneal Dialysis.

**Table 3:** Domains and example questions from the survey of Latin American experts on educational quality and learning assessment

Domain	Example question	Question type
Current state of PD education and gaps in educational quality	Does a formal PD education programme currently exist in your country for doctors and nurses?	Closed (yes/no)
Current state of PD education and gaps in educational quality	Have significant gaps in the educational quality of nephrology staff been identified in your country?	Likert (1–5)
Educational theory, learning assessment, and the doctor’s role in PD education	Does your country’s healthcare system allow for the implementation of education programmes based on andragogy?	Closed (yes/no)
Educational theory, learning assessment, and the doctor’s role in PD education	Are current evaluation methods for PD programmes appropriate and effective?	Likert (1–5)
Evaluation of educational programmes and the doctor’s role in quality improvement	Does the doctor responsible for PD actively participate in the review and updating of educational programmes?	Closed (yes/no)
Evaluation of educational programmes and the doctor’s role in quality improvement	Should the doctor be an integral part of the design and improvement of patient training programmes in PD?	Likert (1–5)

Abbreviation – PD: Peritoneal Dialysis.

**Table 4:** Domains and example questions from the survey of Latin American experts on peritoneal dialysis access

Domain	Example question	Question type
Economic barriers	What are the main economic barriers patients face in accessing PD in your country?	Open-ended
Transportation and logistics costs	How do transportation and logistics costs affect the availability of PD in rural and urban areas?	Open-ended
Financial concerns	What are the main financial concerns patients have when choosing PD?	Open-ended
Psychosocial indicators	What psychosocial indicators should be defined to monitor quality PD treatment?	Open-ended
Nutritional indicators	What nutritional indicators should be defined to monitor the quality of PD treatment?	Open-ended

Abbreviation – PD: Peritoneal Dialysis.

### Working Groups and Debate (1st Delphi Round)

Following the analysis of the surveys, thematic working groups were organised on education, public policies and regulations, and access to PD. These groups served as the first round of the Delphi process, during which initial results were debated, priorities were identified, and preliminary proposals were formulated.

On the first day, participants thoroughly discussed the survey conclusions. This stage allowed for a critical review of expert viewpoints and a first informal round of voting on the proposals.

An iteration and feedback based on the group results were provided, allowing participants to adjust their proposals and priorities.

### Plenary Session for Final Consensus (2nd Delphi Round)

On day 2, a plenary session was organised functioning as the second Delphi round, during which the proposals discussed in the working groups were reviewed and consolidated. Participants had the opportunity to re-evaluate their opinions based on the consensus reached during the working groups.

At this stage, a final consensus was reached on the three main axes (education, public policies and regulations, and access), which allowed for the development of a detailed proposal document tailored to the local realities of each country.

### Validation and Signing of the Declaration (Delphi Validation)

Once consensus was reached, the results were formalised in the Santo Domingo Declaration, which included specific commitments to implement the agreed measures in each country. This document was signed by the participating representatives, who committed to carrying out the proposed actions.

This structured, iterative, and data-driven approach, similar to the Delphi method, allowed for the involvement of multiple key stakeholders, resulting in a robust and representative consensus on how to improve PD in Latin America.

## Results

### Education

The data below present the domains identified during the Education Working Group at the Latin American Workshop on Peritoneal Dialysis Access. These domains cover fundamental aspects of improving PD education, from clinical staff training to the creation of educational platforms for patients. Data from the survey of participating experts and the learning assessment survey of Latin American nephrologists conducted by LACDD-SLANH were analysed (Table 5).

**Table 5:** Results of the survey of Latin American experts on education

Domain	Finding
Clinical staff training	Training in PD is insufficient compared with haemodialysis and transplantation, leading to an imbalance in competencies.
Organisation of educational programmes	There is a lack of formal accreditation for health educators, which limits the implementation of effective programs.
Learning assessment	A lack of standardisation in learning assessment makes the objective measurement of competencies difficult.
Physician’s role as auditor of patient education programmes	The physician’s role as an auditor of educational programmes is not standardised, affecting effective oversight.
Use of advanced educational technologies	There is limited use of advanced technologies, restricting access to personalised and effective educational resources.
Minimum training criteria	The lack of standardisation in PD training programmes creates gaps in educational quality.
Online patient school	Patient education in PD is not accessible or standardised across the region, impacting adherence.

Abbreviation – PD: Peritoneal Dialysis.

The analysis of these findings reveals the urgent need to standardise PD training programmes in Latin America, both for healthcare professionals and for patients. The lack of adequate PD training, the lack of accreditation for educators, and the poor standardisation of learning assessment create a significant gap in care quality. Furthermore, the limited use of advanced educational technologies restricts access to innovative resources that could improve adherence and outcomes in PD treatment.

The survey conducted with nephrologists in Latin America to evaluate the level of learning in key areas of PD included closed (yes/no) questions and Likert-type questions. The identified critical points are those that did not meet formative expectations, that is, responses reflecting areas of opportunity or insufficiency in learning and clinical practice.

The critical points reflect the proportion of respondents who, according to their answers, did not reach the expected levels of training. Likert-type responses with scores from 1 to 3 were considered critical, as they did not meet expectations. The total number of respondents (n), the proportion of critical responses (%), and the absolute number (n of the percentage) representing these critical responses are presented.

As shown in Table 6, several important conclusions can be drawn regarding the learning and competencies of nephrologists in Latin America in relation to PD. The data show several critical points that indicate areas in which formative expectations are not being met:

– Research planning within the ethical and legal framework: This is one of the most critical points, with 68.78% of respondents not meeting expectations. This reflects the need for greater training in research and knowledge of ethical regulations.
– Evaluation of structure, process, and outcome indicators of the PD centre: with 59.12% critical responses, nephrologists show difficulties in evaluating indicators, which are fundamental for the efficient management of PD centres.
– Performance of continuous ambulatory peritoneal dialysis (CAPD) exchanges (31.5%) and cycler connections (59.4%): significant discrepancies are present, as 31.5% of respondents do not know how to perform a manual CAPD exchange and 59.4% have no experience or knowledge of cycler connection procedures. These are critical practical aspects that directly impact professionals’ ability to provide adequate care.
– Interaction with the multidisciplinary care team: 32.04% report deficiencies in interaction with other professionals, suggesting the need for improvement in teamwork and coordination in the clinical setting.
– Participation in the management of a PD centre (55.52%) and critical analysis of indicators (58.56%): more than half of respondents do not feel prepared to adequately manage PD centres or perform critical analyses of indicators.

**Table 6:** Results of the survey of Latin American nephrologists on learning

Critical points	No. of respondents	Critical proportion (%) (did not meet expectations)	No. of cases (frequency)
Planning of research within the ethical and legal framework	362	68.78%	249
Evaluation of structure, process, and outcome indicators of the PD centre	362	59.12%	214
Critical analysis of indicators	362	58.56%	212
Interaction with the multidisciplinary care team	362	32.04%	116
Participation in the management of a PD centre	362	55.52%	201
Contribution to therapeutic education of patients and caregivers	362	34.25%	124
Performing CAPD exchanges	362	31.50%	114
Performing connections to cyclers	362	59.40%	215

Abbreviations – PD: Peritoneal Dialysis; CAPD: Continuous Ambulatory Peritoneal Dialysis.

### Key Discordant Point

A key aspect that warrants further attention is the therapeutic education of patients. Only 124 respondents (34.25%) stated that they did not meet expectations in this regard, which would seem to indicate that most (238 respondents) actively participate in patient education.

However, these data are contradictory to other fundamental aspects, as almost 60% of respondents have no experience in using cyclers, and 31.5% do not know how to perform a manual CAPD exchange.

This creates a concerning discrepancy: how is it possible that most respondents claim to participate in patient therapeutic education if they do not master essential practical skills such as cycler management or manual exchange? Adequately educating PD patients requires a complete mastery of these techniques. This inconsistency suggests a possible overestimation of respondents’ educational abilities or that patient education programmes do not sufficiently address these critical areas.

### Public Policies

Table 7 illustrates the main results obtained from the survey conducted with 27 nephrologists from 14 Latin American countries, focused on key aspects of PD. These results highlight perceptions regarding the preference for PD as a first-line treatment option, the existence of regulations, economic and geographical barriers, and the need for a robust legislative framework to promote PD.

**Table 7:** Results of the survey of Latin American experts on public policies

Point	Explanation
Preference for PD as the first option	91% of respondents agree or strongly agree that PD is the best option for initiating dialysis in incident patients.
PD legislation	63% of respondents report that their countries have regulations for the development of PD programmes, but only 50% state that these regulations include all necessary aspects.
Informed consent	81% of respondents indicate that there is no law guaranteeing informed consent to ensure that patients are informed about all available treatment options (PD and haemodialysis).
Priority of PD as the first option	Only one country has a law establishing PD as the first treatment option, but there are no mechanisms to ensure its enforcement.
PD financing	38% of countries do not guarantee full funding for PD, and in 25% of countries, co-payment is required from patients.
Regional accessibility	69% of respondents report that PD is not accessible in all regions of their country, suggesting logistical and infrastructure challenges.
Need for a law to prioritize PD	87% of respondents believe there should be a law that prioritises and funds PD as the first treatment option.
Barriers to PD implementation	The main barriers to PD implementation are lack of education and experience, followed by lack of funding and inadequate public policies.

Abbreviation – PD: Peritoneal Dialysis.

The survey results reveal that, while there is broad consensus within the medical community on the benefits of PD as a first-line therapy for incident dialysis patients, there are significant gaps in the implementation and regulation of PD programmes in the respondents’ countries. The lack of comprehensive regulations, the absence of informed consent laws, and the lack of mechanisms to ensure equitable access to PD in all regions are obstacles that need to be addressed.

Furthermore, the survey highlights that insufficient funding and the requirement of co-payments in some countries limit access to this treatment, especially for economically vulnerable patients. Likewise, barriers related to education and experience among medical professionals are identified as key factors hindering the expansion of PD programmes.

Finally, a high percentage of respondents consider the enactment of laws that prioritise and fund PD as a first-line dialytic treatment option to be necessary, suggesting that the implementation of an adequate legislative framework is essential to overcome current barriers and improve access to PD throughout the region.

### Access

Table 8 illustrates the main findings derived from the survey conducted on sustainable and equitable access to PD in Latin America. These findings reflect proposals to improve the accessibility, quality, and cost-effectiveness of PD in the region, based on scientific evidence and respondents’ experiences.

**Table 8:** Results of the survey of Latin American experts on access to peritoneal dialysis

Finding/Idea	Justification
Create PD centres in urban and rural areas to improve accessibility	Increases access to PD in areas where infrastructure is limited, ensuring equitable distribution of services.
Establish satellite units under the supervision of responsible nephrologists	Ensures quality of care and specialised expertise, even in regions with fewer specialists.
Use geographical information systems to identify areas of need and plan expansion	Improves efficiency in resource allocation and planning of new PD units based on population distribution.
Provide scientific costeffectiveness data to facilitate financing decisions	Supports evidencebased decisions that favour PD as a cost-effective and sustainable option compared to haemodialysis.
Create efficient financing models that optimise equity in access to renal replacement therapy	Improves access to PD as a more costeffective therapy, especially in low-resource countries, reducing longterm fiscal impact.
Establish key indicators to monitor the quality and efficiency of PD programmes	Enables continuous and effective monitoring to ensure high standards of care and sustainability are maintained.
Develop standardised protocols for urgent PD, ensuring immediate access to therapy	Ensures patients have safe and effective access to PD in critical situations, improving clinical outcomes.
Establish shortstay units for patients initiating urgent PD	Facilitates patient transition to PD, allowing intensive monitoring during the first days of treatment.
Implement digital platforms and telemedicine to facilitate continuous education and remote monitoring	Improves patient education and access to remote follow-up, optimizing selfcare and adherence to treatment in remote regions.

Abbreviation – PD: Peritoneal Dialysis.

Strategies related to the expansion of PD units, optimisation of funding, and the use of technologies to improve treatment planning and monitoring are highlighted.

The survey findings reveal that the expansion of PD is a viable and cost-effective solution to address disparities in access to renal replacement therapy, especially in rural and hard-to-reach areas. The creation of satellite units under the supervision of nephrologists and the implementation of geographical information systems are key strategies to ensure equitable distribution of services.

Additionally, scientific evidence on the cost-effectiveness of PD vs. haemodialysis should be leveraged to implement more efficient funding models that ensure equitable and sustainable long-term access. The establishment of key indicators to monitor the quality and efficiency of PD programmes will allow for adequate outcome monitoring, ensuring that patients receive high-quality care.

In conclusion, these findings provide clear guidance for strengthening policies and strategic planning related to PD, ensuring that this therapeutic modality becomes a central tool for reducing disparities in renal care in Latin America.

## Discussion

The Santo Domingo Declaration highlights the urgent need to address inequalities in nephrology and peritoneal dialysis (PD) training and care in Latin America, a challenge shared with other regions such as Europe, the United States, and China. Variability in infrastructure and access to resources, coupled with disparities in training, perpetuates unequal and suboptimal care.

In analysing other regions, such as Europe, tools like Capabilities in Practice (CiPs) and Generic Professional Capabilities (GPCs), developed by the General Medical Council (GMC), have been implemented to standardise essential competencies and ensure quality in training.

However, their application varies due to differences in infrastructure and access to advanced resources, similar to those observed in Latin America^11,12^. In the United States, barriers to implementing optimal peritoneal dialysis programmes include educational, regulatory, and financial limitations, affecting both patients and healthcare professionals^13^.

On the other hand, China has made significant progress in PD training through initiatives by the Chinese Society of Nephrology and the creation of centres of excellence that promote homogeneous standards and continuous education. However, inequalities between rural and urban areas persist, impacting training in less developed regions^14^.

Initiatives such as the European proposition to standardise essential competencies offer a valuable model for Latin America, where differences in infrastructure and access are compounded by disparities in training.

Latin America, like other regions, faces significant challenges in the adoption of peritoneal dialysis, marked by regional disparities in its use, patient-centred barriers, and limitations within healthcare centres and health systems, in addition to an excessively mechanistic approach to medical care, which limits the integration of patient-centred objectives, as proposed by the SONG initiative.

PD usage rates in Europe show notable disparities: while Scandinavian countries present adoption rates of up to 35%, in nations such as Greece and Romania, this percentage falls below 5%. Furthermore, only 12% of patients over 75 years old use PD, despite its benefits in this population^15^.

The Standardised Outcomes in Nephrology (SONG) initiative, especially its branch dedicated to peritoneal dialysis (SONG-PD), provides a global framework for standardising PD outcomes and prioritising those that are important to both patients and professionals. However, this initiative does not establish criteria for the training of healthcare teams to achieve these objectives.

Integrating these standards into the region could improve the equity and effectiveness of training programmes, ensuring that professional competencies are aligned with the most relevant clinical outcomes^16^.

In the region, more than 59% of surveyed doctors lack experience in cycler management, and 31.5% do not know how to perform manual PD exchanges. These figures reflect the lack of standardisation in training programmes, a problem that could be addressed by adapting tools such as the European Specialty Examination in Nephrology (ESENeph), which in Europe guarantees the certification of minimum competencies and facilitates professional mobility^17^. These inequalities hinder the harmonisation of standards in nephrologist training. Interpersonal and communication skills, as well as health systems-based practice, are essential to address the complexities of the healthcare environment in the region. The inclusion of mandatory rotations in primary care could also contribute to improving the prevention and early management of kidney diseases^18^.

The impact of these inequalities is not limited to training but also affects the quality of care that patients receive. In the United Kingdom, the Francis Report highlighted how deficiencies in training and supervision may compromise patient safety^19^.

Tools such as the ESENeph could be adapted to the Latin American context to guarantee minimum competencies and facilitate professional mobility among countries^20^.

Regarding public policies, Hong Kong’s experience with the “PD First” policy demonstrates that prioritising PD can be a cost-effective and equitable strategy for treating patients with chronic kidney failure^21^. In Latin America, although 91% of surveyed doctors believe that PD should be the preferred initial therapy, only El Salvador has specific legislation that prioritises it. Financial barriers, such as co-payments, also limit its large-scale adoption^22^.

The lack of equitable access to high-quality therapies, such as PD, reflects a structural inequity in medical care that disproportionately affects vulnerable populations. Integrating a human rights-based approach into renal health policies is fundamental to ensuring that all individuals, regardless of their location or socioeconomic status, can access these therapies. Vanholder et al. highlight that inequities in renal health not only limit access but also perpetuate existing social inequalities^23^.

The creation of a regulatory framework that includes key indicators to monitor quality and access, supported by health ministries, scientific societies, and patient organisations, can contribute to reducing these gaps and ensuring that PD is accessible and affordable for all.

Unequal access to PD also reflects a structural problem. In rural areas, the lack of infrastructure limits the availability of home therapies. In Europe, the development of satellite units supervised by specialised nephrologists and the use of telemedicine have significantly improved access in remote regions^24^. In Latin America, Colombia provides another example, with the impact of programmes in local communities^25^.

One of the problems facing Latin America is the fragmentation of its healthcare systems and how this hinders integrated home care for patients. An interesting initiative has been implemented in Peru with a home visit programme for peritoneal dialysis patients delivered by primary care health personnel and funded by the *Fondo Intangible de Seguridad Social del Ministerio de Salud* ^26^.

### Proposed Actions

Unlike countries where PD access is better structured and standardised, Latin America faces structural problems that must be resolved before advancing towards the standardisation of clinical outcomes. While SONG-PD focuses on priority outcomes such as technical survival, infection reduction, and participation in daily life, in Latin America the organisation of fundamental processes must be prioritised before implementing these standards.

Strengthening training, developing robust public policies, and ensuring equitable access are essential preliminary steps to reduce current inequalities and achieve more efficient implementation of PD in the region.

Table 9 illustrates a set of strategic proposals to improve access, training, and policies related to PD in Latin America. These proposals, organised around the macrodomains of education, public policies, and access, aim to strengthen the health system in the region, promote equity, and ensure the continuous training of medical professionals and patient education.

**Table 9:** Action proposals of the Santo Domingo Declaration

Macrodomain	Proposal	Description
Education	Minimum training criteria	Establish standardised minimum criteria for PD training, aligned with SLANH-LACDD, covering practical competencies such as catheter placement and complication management, applicable both to nephrologists in training and to continuing education.
	Quality seal for training and regional rotation units	Create a quality seal to certify PD training units in Latin America. These units must have qualified staff, adequate infrastructure, and allow regional rotations in specialised centres, providing significant clinical exposure to ensure comprehensive training.
	Advanced online PD course	Develop an advanced online PD course with structured training phases (initial, basic, advanced, and head of service), accredited by SLANH-LACDD and tailored to local needs. This course should include theoretical modules, clinical simulations, and a strong practical component to train professionals at each level.
	Online patient school	Create an online patient school led by SLANH-LACDD to educate patients throughout Latin America in home PD management. The platform will include multimedia materials and accessible resources to reinforce learning at any time, improving treatment adherence and clinical outcomes.
	Development of educational materials and use of advanced technology	Invest in innovative educational materials, such as visual guides, interactive simulations, video tutorials, and the use of artificial intelligence to personalise learning. Technological platforms should include remote monitoring tools and automated reminders for home treatment management.
	Training in health education	Create a training programme for health educators (physicians and nurses) based on andragogy, constructivism, and the use of educational technologies. Professionals should be trained to design meaningful educational programmes tailored to adult PD patients.
	Continuous evaluation of the educational programme	Implement a continuous evaluation system based on rubrics to measure the competencies acquired by patients and staff. These evaluations should be integrated into the management of the PD unit, improving communication between physicians and nurses and ensuring patient progress in educational area.
	Development of standardised training programmes	Design standardised curricula for the training of nephrologists, nurses, and primary care staff, adapted to local needs and focused on PD as the preferred home therapy.
Public policies	Equitable access to renal replacement therapy	Ensure adequate financial and operational conditions in each Latin American country to reduce inequities in access to renal replacement therapies through the implementation of a global health programme starting at a primary care level, with predialysis clinics aimed at supporting informed decision making.
	Priority of PD as home therapy	Establish a regional policy favouring PD as home therapy, with national regulations eliminating the practice of intermittent peritoneal dialysis. This policy should be led by the SLANH-LACDD and the *Organismo Andino de Salud – Convenio Hipólito Unanue*.
	National registries of patients on renal replacement therapies	Create or strengthen national registries of patients receiving renal replacement therapy and those in stages 3 and 4 of chronic kidney disease, to improve planning, followup, and epidemiological assessment in each Latin American country.
	Economic evaluation of renal replacement therapies	Conduct economic evaluation studies of renal replacement therapies to optimise resource allocation and inform policy development, under the coordination of SLANH-LACDD.
	Development of a legal framework for PD	Propose changes to national health policies, designing or updating clinical guidelines that favour the expansion of PD as home therapy, adapting these regulations to the Latin American context.
Access to PD	Formation of a steering committee	Establish a steering committee with representatives from health ministries, nephrology societies, and patient organisations, to define roles and responsibilities in the implementation of the PD programme. This committee will also develop a strategic plan for PD expansion.
	Implementation of satellite PD units and telemedicine	Promote the creation of satellite PD units providing care to local communities, incorporating telemedicine technologies to facilitate remote patient monitoring and consultations, thereby improving access to specialised care in remote areas.
	Expansion and evaluation of the PD programme	Gradually expand the PD programme in Latin American countries based on results from pilot phases. Data on access, clinical outcomes, and costs should be periodically evaluated to adjust the programme and ensure effective and sustainable implementation.
	Integration of PD into universal health systems	Work towards incorporating PD into universal health systems in the region, ensuring PD coverage and its inclusion as the preferred option when appropriate, strengthening integrated care pathways across different levels of care.
Integration of policies and access	International Group for Renal Policies and Access (GIPAR)	The creation of GIPAR is proposed – a public policy support group composed of representatives from SLANH LACDD, the *Organismo Andino de Salud – Convenio Hipólito Unanue*, and nephrology societies from each country. GIPAR will coordinate with local governments, develop proposals to improve access to renal replacement therapies, and ensure the implementation of regulations favouring PD as the preferred home therapy. GIPAR will work towards establishing a common regulatory framework based on best practices, adapted to each country, promoting equity and universal access to renal replacement therapy in the region.
		Coordinators: Dr Rosana Chaúd Covarrubias and Dr Laura Cortés.

Abbreviations – PD: Peritoneal Dialysis; LACDD: Latin American Chapter of Home Dialysis; SLANH: Latin American Society of Nephrology and Hypertension.

Each proposal has been designed to address current gaps in chronic kidney disease care, from the implementation of training programmes to the creation of international teams to drive necessary public policies. Collaboration between SLANH-LACDD, the *Organismo Andino de Salud – Convenio Hipólito Unanue*, and the nephrology societies of each country will be key to the successful execution of these initiatives.

## Conclusions

The Santo Domingo Declaration marks a milestone in improving PD in Latin America. However, for the commitments detailed here to become effective actions, collaboration between scientific societies, health ministries, and universities in each country is fundamental.

Only through a combined effort can appropriate public policies be developed, healthcare professionals’ training be optimised, and equitable access to essential treatments be ensured. Therefore, we propose the creation of national working groups in which these key stakeholders can identify local barriers and adapt the strategies of the Santo Domingo Declaration to the specific needs of each region. We invite the leaders of these institutions to join intersectoral meetings that will allow progress towards a more inclusive and efficient health system for all patients with chronic kidney disease in Latin America.

## Data Availability

The datasets generated and/or analysed during the current study are available from the corresponding author upon reasonable request.

## References

[1] Organización Mundial de la Salud (2019). El acceso desigual a los servicios de salud genera diferencias en la esperanza de vida [Internet]. https://www.who.int.

[2] Morton RL, Shah KK (2021). Kidney health in the context of economic development. Nat Rev Nephrol.

[3] Stanifer JW, Muiru A, Jafar TH, Patel UD (2016). Chronic kidney disease in low- and middle-income countries. Nephrol Dial Transplant.

[4] Comisión Económica para América Latina y el Caribe (2016). La matriz de la desigualdad social en América Latina [Internet].

[5] Di Bernardo JJ, González-Martínez F, Di Rienzo P, Cortés-Sanabria L, Cerdas-Calderón M, García-García G (2016). Caracterización de los programas de especialización en nefrología de América Latina. FEM.

[6] Li PK, Chow KM (2013). Peritoneal dialysis-first policy made successful: perspectives and actions. Am J Kidney Dis.

[7] Manera KE, Tong A, Craig JC, Brown EA, Brunier G, Dong J (2017). Standardized Outcomes in Nephrology–Peritoneal Dialysis (SONG-PD): study protocol for establishing a core outcome set in PD. Perit Dial Int.

[8] Divino J, Moretta GM, Marcos S, Chaud R, Bastos K, Arias Minaya C, Fadem SZ, Moura JA (2021). Issues in kidney disease – dialysis.

[9] Wong CK, Chen J, Fung SK, Mok M, Cheng YL, Kong I (2020). Lifetime cost-effectiveness analysis of first-line dialysis modalities for patients with end-stage renal disease under peritoneal dialysis first policy. BMC Nephrol.

[10] Varela-Ruiz M, Díaz-Bravo L, García-Durán R (2012). Descripción y usos del método Delphi en investigaciones del área de la salud. Investig Educ Med.

[11] General Medical Council (2013). Good medical practice [Internet]. https://www.gmc-uk.org.

[12] General Medical Council (2017). Generic professional capabilities framework [Internet]. https://www.gmc-uk.org.

[13] Finkelstein FO (2020). Barriers to optimal peritoneal dialysis. Semin Dial.

[14] Zhang L, Zuo L (2022). Advances in peritoneal dialysis in China: training programs and policies for growth. Kidney Int Suppl (2011).

[15] Wong CK, Chen J, Fung SK, Mok M, Cheng YL, Kong I (2020). Lifetime cost-effectiveness analysis of first-line dialysis modalities for patients with end-stage renal disease under peritoneal dialysis first policy. BMC Nephrol.

[16] Di Bernardo JJ, González-Martínez F, Di Rienzo P, Cortés-Sanabria L, Cerdas-Calderón M, García-García G (2016). Caracterización de los programas de especialización en nefrología de América Latina. Educ Med.

[17] Zhang L, Zuo L (2022). Advances in peritoneal dialysis in China: training programs and policies for growth. Kidney Int Suppl (2011).

[18] Manera KE, Tong A, Craig JC, Brown EA, Brunier G, Dong J (2017). Standardized Outcomes in Nephrology–Peritoneal Dialysis (SONG-PD): establishing a core outcome set in peritoneal dialysis. Perit Dial Int.

[19] Joint Royal Colleges of Physicians Training Board (2022). European Specialty Examination in Nephrology (ESENeph) [Internet]. https://www.mrcpuk.org.

[20] Brown EA, Boni Brivio G, Van Biesen W (2024). Towards a better uptake of home dialysis in Europe: understanding the present and looking to the future. Clin Kidney J.

[21] Di Bernardo JJ, González-Martínez F, Di Rienzo P, Cortés-Sanabria L, Cerdas-Calderón M, García-García G (2016). Caracterización de los programas de especialización en nefrología de América Latina. Educ Med.

[22] Francis R (2013). Report of the Mid Staffordshire NHS Foundation Trust Public Inquiry [Internet]. https://www.gov.uk/government/publications/report-of-the-mid-staffordshire-nhs-foundation-trust-public-inquiry.

[23] Vanholder R, Luyckx VA, Hoekstra T, Wanner C, Massy ZA, Herz D (2023). Inequities in kidney health and kidney care: a global perspective. Nat Rev Nephrol.

[24] Wong CK, Chen J, Fung SK, Mok M, Cheng YL, Kong I (2020). Lifetime cost-effectiveness analysis of first-line dialysis modalities for patients with end-stage renal disease under peritoneal dialysis first policy. BMC Nephrol.

[25] Sanabria M, Mitchell H, Rosner J, Vesga J, Molano A, Corzo L (2018). A remote management program in automated peritoneal dialysis patients in Colombia. Nefrol Latinoam.

[26] Seguro Integral de Salud (2022). Fondo Intangible Solidario de Salud. Convenio entre SIS-FISSAL y GORE Lima. Periodo 2022-2024 [Internet].

