# Supplementary material for: Declaration of Santo Domingo: commitment to improve education, regulations, public policies, and access to peritoneal dialysis in Latin America
Source: J Bras Nefrol. 2026 Apr 3;48(2):e2026SA01. doi: 10.1590/2175-8239-JBN-2026-SA01en (PMC13078817; doi:10.1590/2175-8239-JBN-2026-SA01en)
Supplement: Translation

# Translation: Declaração de Santo Domingo: compromisso com a melhoria da educação, das regulamentações, das políticas públicas e do acesso à diálise peritoneal na América Latina

**DOI:** 10.1590/2175-8239-JBN-2026-SA01pt

**Authors:** Gustavo L. Moretta-Pedernera, Alfonso Ramos-Sánchez, Laura Cortés-Sanabria, Rosana Chaúd-Covarrubias, Guillermo Álvarez-Estévez, Mauricio Uribe, Vicente Sánchez-Polo, José Carolino Divino-Filho, Natalia M. Wasmuth-Troche, Ledisbeth Batista-Buitrago, Marta Avellán-Boza, Rodrigo Orozco-Bass, Héctor Martínez-Eusebio, René Soto-Velásquez, María Marina Papaginovic-Leiva, Martín Gómez-Luján, Sandra Rodríguez-Manzano, José Manuel Rodríguez-Chagolla, Daniel Alejandro Molina-Comboni, Yanet Álvarez-González, Pedro Javier Dávila Crespo, Erwin Iván Campos, José A. Moura-Neto, Magda Hinojosa, Lourdes Carolina Vásquez-Jiménez, Gloria G. García-Villalobos, Gabriela González, Eliana Dina-Batle, José R. Álvarez-Novoa

**Affiliations:** 1 Capítulo Latinoamericano de Diálisis Domiciliaria (LACDD), Latin America.; 2 Universidad de San Andrés, Buenos Aires, Argentina.; 3 Silher Corporación, Lima, Perú.; 4 Macrotech Farmacéutica, Mexico City, México.; 5 Sociedad Latinoamericana de Nefrología e Hipertensión (SLANH), Latin America.; 6 Universidad de Guadalajara, Centro Universitario de Tonalá, Jalisco, México.; 7 Fondo Intangible Solidario de Salud, Ministerio de Salud, Lima, Perú.; 8 CEDIMAT, Santo Domingo, República Dominicana.; 9 Asociación Colombiana de Nefrología e Hipertensión, Bogotá, Colombia.; 10 Universidad Pontificia Bolivariana, Medellín, Colombia.; 11 Universidad de San Carlos, Ciudad de Guatemala, Guatemala.; 12 Karolinska Institutet, Stockholm, Sweden.; 13 Sociedad Paraguaya de Nefrología, Asunción, Paraguay.; 14 Hospital Central del Instituto de Previsión Social, Asunción, Paraguay.; 15 Sociedad Panameña de Nefrología e Hipertensión, Panama City, Panamá.; 16 Hospital Regional Rafael Hernández, Chiriquí, Panamá.; 17 Asociación Costarricense de Nefrología, San José, Costa Rica.; 18 Universidad de Costa Rica, San José, Costa Rica.; 19 Hospital Enrique Baltodano, Liberia, Guanacaste, Costa Rica.; 20 Sociedad Chilena de Nefrología, Santiago, Chile.; 21 Universidad de los Andes, Santiago, Chile.; 22 Sociedad Dominicana de Nefrología, Santo Domingo, República Dominicana.; 23 Hospital Dr. Francisco Moscoso Puello, Santo Domingo, República Dominicana.; 24 Asociación de Nefrología e Hipertensión Arterial El Salvador, San Salvador, El Salvador.; 25 Instituto Salvadoreño del Seguro Social, San Salvador, El Salvador.; 26 Centro de Hemodiálisis de El Salvador, San Salvador, El Salvador.; 27 Sociedad Argentina de Nefrología, Buenos Aires, Argentina.; 28 Universidad Católica Argentina, Buenos Aires, Argentina.; 29 Complejo Médico Churruca-Visca, Buenos Aires, Argentina.; 30 Sociedad Peruana de Nefrología, Lima, Perú.; 31 Universidad Nacional Federico Villarreal, Lima, Perú.; 32 Hospital Regional Antonio Musa, San Pedro de Macorís, República Dominicana.; 33 Colegio de Nefrólogos de México A.C., Mexico City, México.; 34 Universidad Nacional Autónoma de México, Mexico City, México.; 35 Centro Médico ISSEMYM, Toluca, México.; 36 Sociedad Boliviana de Nefrología, La Paz, Bolivia.; 37 Hospital Guaracachi, Santa Cruz, Bolivia.; 38 Caja Petrolera de Salud, Santa Cruz, Bolivia.; 39 Clínica Incor, Santa Cruz, Bolivia.; 40 Sociedad Cubana de Nefrología, Havana, Cuba.; 41 Instituto de Nefrología, Havana, Cuba.; 42 Asociación Guatemalteca de Nefrología, Guatemala City, Guatemala.; 43 Unidad Nacional de Atención al Enfermo Renal Crónico, Guatemala City, Guatemala.; 44 Pontificia Universidad Católica Madre y Maestra, Santo Domingo, República Dominicana.; 45 Hospital Salvador Bienvenido Gautier, Santo Domingo, República Dominicana.; 46 Sociedade Brasileira de Nefrologia, São Paulo, Brasil.; 47 Escola Bahiana de Medicina e Saúde Pública, Salvador, Brasil.; 48 Comité Andino de Salud Renal, Andean Region.; 49 Organismo Andino de Salud – Convenio Hipólito Unanue (ORAS-CONHU), Lima, Perú.; 50 Universidad Nacional de Asunción, Asunción, Paraguay.; 51 Instituto Mexicano de Investigaciones Nefrológicas, Mexico City, México.; 52 Hospital General de México Dr. Eduardo Liceaga, Mexico City, México.; 53 Universidad de Buenos Aires, Buenos Aires, Argentina.; 54 Hospital de Clínicas José de San Martín, Buenos Aires, Argentina.; 55 Hospital Metropolitano de Santiago, Santiago, República Dominicana.; 56 Sociedad Nicaragüense de Nefrología, Managua, Nicaragua.; 57 Universidad Nacional Autónoma de Nicaragua, Managua, Nicaragua.; 58 Hospital Bautista, Managua, Nicaragua.; 59 Hospital Escuela Lenin Fonseca Martínez, Managua, Nicaragua.

**Keywords:** Diálise Peritoneal, Declaração de Santo Domingo, Educação Médica, Harmonização, Padrões Internacionais, SONG-PD, ESENeph, Política Pública, Acesso Equitativo, Saúde Renal

## Abstract

A Declaração de Santo Domingo destaca os desafios e as oportunidades para aprimorar a educação, as regulamentações, as políticas públicas e o acesso à diálise peritoneal (DP) na América Latina. Enfatiza a necessidade de enfrentar desigualdades estruturais na formação e na assistência, estabelecendo padrões mínimos de competências e fortalecendo a infraestrutura de saúde. Inspirada em iniciativas como o *Standardized Outcomes in Nephrology–Peritoneal Dialysis* (SONG-PD) e o *European Specialty Examination in Nephrology* (ESENeph), a declaração ressalta a importância da harmonização na formação dos nefrologistas, promovendo uma capacitação uniforme e alinhada aos padrões internacionais. Além disso, aborda estratégias para priorizar a qualidade de vida dos pacientes, garantir acessibilidade e sustentabilidade econômica, e incentivar a implementação de unidades satélites e tecnologias inovadoras. Por fim, faz um chamado à colaboração intersetorial para assegurar um cuidado inclusivo e equitativo na região.

## Introdução

O Workshop Latino-Americano sobre Acesso à Diálise Peritoneal, organizado pela Sociedade Latino-Americana de Nefrologia e Hipertensão (SLANH) e pelo Capítulo Latino-Americano de Diálise Domiciliar (LACDD), reuniu um grupo de especialistas em nefrologia, representantes de sociedades científicas e autoridades de saúde em Santo Domingo, República Dominicana. O objetivo foi discutir os desafios enfrentados pela região em relação ao acesso, às regulamentações, às políticas públicas e à educação em diálise peritoneal (DP).

## Contexto Regional da Doença Renal Crônica

A doença renal crônica (DRC) é uma pandemia silenciosa na América Latina, impulsionada pelo aumento contínuo de condições como diabetes mellitus e hipertensão arterial, que elevam a demanda por terapias renais substitutivas, incluindo a DP. Apesar de seu reconhecido custo-benefício e dos resultados clínicos favoráveis, apenas 12% dos pacientes na região têm acesso a essa modalidade de tratamento.

De acordo com a Organização Mundial da Saúde (OMS), a cobertura universal de saúde implica que todas as pessoas tenham acesso a serviços de saúde de qualidade sem enfrentar dificuldades financeiras, especialmente aquelas pertencentes aos grupos mais vulneráveis. No entanto, na América Latina, as disparidades no acesso à saúde, baseadas em fatores socioeconômicos, demográficos e étnicos, contribuem para diferenças significativas na expectativa de vida e na qualidade do cuidado^1^. A falta de cobertura gera iniquidades em saúde, que podem ser corrigidas por meio de políticas adequadas^2^.

Na região, quase um terço da população relata não buscar atendimento médico mesmo quando necessita. Barreiras financeiras, escassez de recursos (como profissionais de saúde e medicamentos) e dificuldades geográficas agravam o cenário. Essas barreiras são particularmente severas nos quintis mais pobres da população, evidenciando a inequidade estrutural no acesso à assistência médica — incluindo a diálise peritoneal^3^.

## Desafios e Oportunidades Para a Diálise Peritoneal

As regulamentações na América Latina são heterogêneas em relação à organização e à administração dos centros de diálise peritoneal, impactando diretamente tanto na qualidade do tratamento oferecido quanto na expansão dessa terapia na região^4^. Além disso, os programas de especialização em nefrologia apresentam marcada heterogeneidade em sua duração e carga horária, variando de 2 a 5 anos, bem como em suas estruturas curriculares, o que dificulta a padronização da formação^5^.

Experiências internacionais têm demonstrado o sucesso da implementação da DP como primeira opção terapêutica, conhecida como política *PD First*. Em países como a Tailândia e Hong Kong^6^, essa estratégia mostrou-se eficaz em melhorar significativamente a cobertura de pacientes com doença renal crônica. Em Hong Kong, por exemplo, a política *PD First* resultou em aumento da sobrevida dos pacientes, além de redução de custos em comparação à hemodiálise. Na Tailândia, a implementação dessa política favoreceu um acesso mais equitativo à terapia renal, tanto em áreas rurais quanto em áreas urbanas, contribuindo para a expansão nacional do tratamento e ajudando a reduzir as listas de espera para hemodiálise.

## Padronização Dos Desfechos em Diálise Peritoneal: Iniciativas Globais e o Contexto Latino-Americano

Em nível global, iniciativas como o *Standardised Outcomes in Nephrology–Peritoneal Dialysis (SONG-PD)* ^7^ demonstraram a importância de se estabelecer um conjunto padronizado de desfechos-chave para a prática clínica em DP. Esse projeto envolve uma ampla gama de atores interessados — incluindo pacientes, cuidadores, clínicos e outros profissionais — que participam da identificação de prioridades compartilhadas, assegurando que os desfechos relatados sejam significativos e relevantes para a tomada de decisão clínica e política.

Entretanto, na América Latina, antes de avançar em direção à padronização de desfechos, como proposto pelo SONG-PD, é fundamental que os países da região enfrentem desafios estruturais básicos. É necessário reorganizar e fortalecer processos essenciais, o que inclui melhorar a capacitação tanto de profissionais de saúde quanto de pacientes, garantindo que estejam adequadamente treinados para o uso da DP. Além disso, é crucial implementar políticas públicas robustas e transparentes, acompanhadas de marcos regulatórios claros, que sustentem a expansão e o acesso equitativo a essa modalidade terapêutica.

Um aspecto central é definir claramente as competências que os egressos dos programas de especialização em nefrologia devem desenvolver, uma vez que muitos programas não o fazem explicitamente, o que limita a clareza do perfil profissional.

No que se refere à educação do paciente, o uso de ferramentas de avaliação, como rubricas, pode ser fundamental para garantir que os pacientes adquiram as competências necessárias para gerenciar seu tratamento em casa. Essas ferramentas permitem uma avaliação objetiva do progresso do paciente em áreas como biossegurança, manuseio da conectologia e gestão de complicações, aspectos cruciais para a melhoria dos desfechos clínicos^8^. A padronização de desfechos, embora seja um objetivo de médio e longo prazo, não pode ser implementada de forma eficaz sem uma infraestrutura adequada que sustente esses esforços. Isso requer uma abordagem abrangente, que enfrente tanto a escassez de recursos humanos especializados quanto as desigualdades no acesso às terapias renais, problemas ainda persistentes em grande parte da América Latina.

A experiência de países como a Tailândia e Hong Kong demonstra que é possível melhorar tanto a cobertura quanto os resultados clínicos quando políticas estruturais são implementadas, priorizando a DP como primeira opção terapêutica^9^.

Esta declaração busca estabelecer um consenso regional para aprimorar a educação, as regulamentações, as políticas públicas e o acesso à diálise peritoneal, garantindo que essas bases estruturais sejam fortalecidas antes de avançar rumo a padrões globais, como os propostos pelo SONG-PD.

Durante o workshop, foram analisadas as principais barreiras, sendo propostas estratégias para aprimorar as regulamentações, as políticas públicas, o acesso e a educação em DP na América Latina. Os signatários desta declaração representam um consenso sobre a necessidade urgente de implementar ações concretas fundamentadas nesses pilares.

## Metodologia de Consenso (Método Delphi)^10^

Para alcançar um consenso sobre como aprimorar a educação, as regulamentações, as políticas públicas e o acesso à diálise peritoneal na América Latina, foi utilizada uma abordagem metodológica inspirada no Método Delphi, adaptada às necessidades e às características regionais. Essa abordagem permitiu uma participação estruturada, iterativa e colaborativa de especialistas em nefrologia e representantes de diversas sociedades científicas. Os principais elementos do processo são descritos a seguir.

### Levantamentos Iniciais Com Nefrologistas e Representantes de Sociedades Científicas

O processo iniciou-se com a coleta de dados por meio de duas pesquisas:

– Pesquisa com nefrologistas: direcionada a 362 nefrologistas latino-americanos, com o objetivo de avaliar o estado atual da educação em DP, identificar lacunas de formação e medir a implementação de abordagens pedagógicas modernas, como a andragogia, além de avaliar suas práticas clínicas. A Tabela 1 apresenta a classificação dos principais domínios avaliados na pesquisa sobre DP, juntamente com exemplos de perguntas para cada domínio e o tipo de questão utilizada. Foram empregados dois tipos de questões: fechadas, que buscam respostas concretas e específicas, e tipo *Likert*, que permitem aos respondentes autoavaliar seu grau de confiança ou preparo em diferentes aspectos da formação. Essa combinação de formatos fornece tanto dados objetivos quanto uma compreensão qualitativa da experiência educacional em DP.
– Pesquisa com representantes de sociedades científicas: participaram 27 representantes, com o objetivo de coletar informações sobre educação, regulamentações, políticas e acesso à DP. As Tabelas 2 a 4 detalham os principais domínios avaliados. Por meio de questões fechadas, tipo *Likert*, e abertas, foram reunidas opiniões sobre regulamentações, cumprimento de políticas públicas, financiamento e barreiras enfrentadas pelos programas de DP. Essa análise proporciona uma visão abrangente das áreas que necessitam de ação e aprimoramento para garantir melhor acesso à DP na América Latina. As Tabelas 2 a 4 apresentam exemplos das perguntas realizadas e o tipo de questão utilizado em cada domínio.

**Tabela 1:** Domínios e exemplos de perguntas da pesquisa com nefrologistas latino-americanos

Domínio	Exemplo de pergunta	Tipo de pergunta
Dados demográficos e formação	Em que ano você obteve o título de especialista em nefrologia?	Fechada
Experiência em DP	Quantas horas você dedicou à DP em comparação à hemodiálise durante sua formação?	Fechada
Procedimentos e habilidades técnicas	Você realizou implante de cateteres peritoneais durante sua formação?	Fechada
Participação em atividades de gestão e avaliação	Você participou de reuniões clínicas com a equipe multidisciplinar?	Fechada
Formação complementar	Você realizou cursos de DP fora do seu programa de pós-graduação?	Fechada
Experiência em DP	Quão competente você se sente para prescrever DPAC/DPA?	*Likert*
Procedimentos e habilidades técnicas	Quão competente você se sente no manejo de complicações infecciosas?	*Likert*
Participação em atividades de gestão e avaliação	Quão competente você se sente na interação com a equipe multidisciplinar?	*Likert*

Abreviaturas – DP: Diálise Peritoneal; DPA: Diálise Peritoneal Automatizada; DPAC: Diálise Peritoneal Ambulatorial Contínua.

**Tabela 2:** Domínios e exemplos de perguntas da pesquisa com especialistas latino-americanos sobre legislação e políticas públicas

Domínio	Exemplo de pergunta	Tipo de pergunta
Regulamentações sobre DP	Você acredita que a DP é a melhor opção para iniciar a diálise em pacientes incidentes?	*Likert* (1–5)
Políticas públicas para priorização da DP	O seu país possui alguma regulamentação para o desenvolvimento de programas de DP?	Fechada (sim/não/talvez)
Acessibilidade e financiamento da DP	No seu país, todas as pessoas têm direito ao financiamento da DP?	Fechada (sim/não/talvez)
Proposta de marco regulatório	Você acredita que a existência de regulamentações favoreceria o desenvolvimento da DP como primeira opção dialítica?	Fechada (sim/não)
Barreiras e ações para implementação da DP	Quais você acredita serem as duas principais barreiras para implementar programas de DP em seu país?	Aberta

Abreviatura – DP: Diálise Peritoneal.

**Tabela 3:** Domínios e exemplos de perguntas da pesquisa com especialistas latino-americanos sobre qualidade educacional e avaliação de aprendizado

Domínio	Exemplo de pergunta	Tipo de pergunta
Estado atual da educação em DP e lacunas na qualidade educacional	Existe atualmente em seu país um programa formal de educação em DP para médicos e enfermeiros?	Fechada (sim/não)
Estado atual da educação em DP e lacunas na qualidade educacional	Foram identificadas lacunas significativas na qualidade educacional da equipe de nefrologia em seu país?	*Likert* (1–5)
Teoria educacional, avaliação da aprendizagem e papel do médico na educação em DP	O sistema de saúde do seu país permite a implementação de programas educacionais baseados em andragogia?	Fechada (sim/não)
Teoria educacional, avaliação da aprendizagem e papel do médico na educação em DP	Os métodos atuais de avaliação dos programas de DP são apropriados e eficazes?	*Likert* (1–5)
Avaliação dos programas educacionais e papel do médico na melhoria da qualidade	O médico responsável pela DP participa ativamente da revisão e atualização dos programas educacionais?	Fechada (sim/não)
Avaliação dos programas educacionais e papel do médico na melhoria da qualidade	O médico deve ser parte integrante do desenho (elaboração) e da melhoria dos programas de treinamento de pacientes em DP?	*Likert* (1–5)

Abreviatura – DP: Diálise Peritoneal.

**Tabela 4:** Domínios e exemplos de perguntas da pesquisa com especialistas latino-americanos sobre acesso à diálise peritoneal

Domínio	Exemplo de pergunta	Tipo de pergunta
Barreiras econômicas	Quais são as principais barreiras econômicas enfrentadas pelos pacientes para acessar a DP em seu país?	Aberta
Custos de transporte e logística	Como os custos de transporte e logística afetam a disponibilidade da DP em áreas rurais e urbanas?	Aberta
Preocupações financeiras	Quais são as principais preocupações financeiras dos pacientes ao optar pela DP?	Aberta
Indicadores psicossociais	Quais indicadores psicossociais devem ser definidos para monitorar a qualidade do tratamento em DP?	Aberta
Indicadores nutricionais	Quais indicadores nutricionais devem ser definidos para monitorar a qualidade do tratamento em DP?	Aberta

Abreviatura – DP: Diálise Peritoneal.

### Grupos de Trabalho e Debate (1ª Rodada Delphi)

Após a análise dos levantamentos, foram organizados grupos de trabalho temáticos sobre educação, políticas públicas e regulamentações, e acesso à diálise peritoneal (DP). Esses grupos constituíram a primeira rodada do processo Delphi, na qual os resultados iniciais foram debatidos, as prioridades foram identificadas e as propostas preliminares foram formuladas.

No primeiro dia, os participantes discutiram detalhadamente as conclusões das pesquisas, permitindo uma revisão crítica das opiniões dos especialistas e uma primeira votação informal sobre as propostas apresentadas.

Em seguida, foi realizado um processo de iteração e feedback com base nos resultados dos grupos, possibilitando aos participantes ajustar suas propostas e redefinir prioridades.

### Sessão Plenária Para Consenso Final (2ª Rodada Delphi)

No segundo dia, foi organizada uma sessão plenária, que funcionou como a segunda rodada Delphi, na qual as propostas discutidas nos grupos de trabalho foram revisadas e consolidadas. Durante essa etapa, os participantes tiveram a oportunidade de reavaliar suas opiniões à luz do consenso alcançado nos grupos de trabalho.

Nessa etapa, foi alcançado um consenso final sobre os três eixos principais — educação, políticas públicas e regulamentações e acesso — o que possibilitou a elaboração de um documento de proposta detalhado, adaptado às realidades locais de cada país.

### Validação e Assinatura da Declaração (Validação Delphi)

Após o alcance do consenso, os resultados foram formalizados na Declaração de Santo Domingo, que incluiu compromissos específicos para a implementação das medidas acordadas em cada país. Esse documento foi assinado pelos representantes participantes, que se comprometeram a executar as ações propostas.

Essa abordagem estruturada, iterativa e baseada em dados, semelhante ao método Delphi, permitiu o envolvimento de múltiplos atores-chave, alcançando um consenso robusto e representativo sobre as melhorias necessárias para a diálise peritoneal na América Latina.

## Resultados

### Educação

Os dados a seguir apresentam os domínios identificados durante o Grupo de Trabalho de Educação no Workshop Latino-Americano sobre Acesso à Diálise Peritoneal. Esses domínios abrangem aspectos fundamentais para o aprimoramento da educação em DP, desde a formação da equipe clínica até a criação de plataformas educacionais para pacientes. Foram avaliados os dados da pesquisa com especialistas participantes e da avaliação de aprendizado dos nefrologistas latino-americanos conduzida pelo LACDD–SLANH (Tabela 5).

**Tabela 5:** Resultados da pesquisa com especialistas latino-americanos sobre educação

Domínio	Achado
Treinamento da equipe clínica	O treinamento em DP é insuficiente em comparação com a hemodiálise e com o transplante, gerando um desequilíbrio nas competências.
Organização dos programas educacionais	Há falta de acreditação formal para educadores em saúde, o que limita a implementação de programas eficazes.
Avaliação da aprendizagem	A ausência de padronização na avaliação da aprendizagem dificulta a mensuração objetiva das competências.
Papel do médico como auditor dos programas de educação de pacientes	O papel do médico como auditor dos programas educacionais não é padronizado, comprometendo a supervisão efetiva.
Uso de tecnologias educacionais avançadas	O uso de tecnologias educacionais avançadas é limitado, restringindo o acesso a recursos personalizados e eficazes.
Critérios mínimos de capacitação	A falta de padronização nos programas de formação em DP gera lacunas na qualidade educacional.
Escola on-line para pacientes	A educação de pacientes em DP não é acessível nem padronizada na região, impactando negativamente a adesão.

Abreviatura – DP: Diálise Peritoneal.

A análise desses achados revela a necessidade urgente de padronizar os programas de capacitação em DP na América Latina, tanto para profissionais de saúde quanto para pacientes. A ausência de formação adequada, a falta de acreditação para educadores e a baixa padronização na avaliação do aprendizado criam uma lacuna significativa na qualidade do cuidado. Além disso, o uso limitado de tecnologias educacionais avançadas restringe o acesso a recursos inovadores que poderiam melhorar a adesão e os desfechos do tratamento em DP.

A pesquisa realizada com nefrologistas latino-americanos para avaliar o nível de aprendizado em áreas-chave da DP incluiu questões fechadas (sim/não) e questões do tipo *Likert*. Os pontos críticos identificados correspondem às áreas que não atenderam às expectativas formativas, ou seja, respostas que refletem oportunidades de melhoria ou insuficiências no aprendizado e na prática clínica.

Esses pontos críticos representam a proporção de respondentes que, segundo suas respostas, não alcançaram o nível esperado de capacitação. No caso das questões do tipo *Likert*, foram consideradas críticas as respostas com pontuação entre 1 e 3. O número total de respondentes (n), a proporção de respostas críticas (%) e o número absoluto (n correspondente) são apresentados.

A partir da Tabela 6, podem ser extraídas conclusões importantes sobre o aprendizado e as competências dos nefrologistas latino-americanos em relação à DP. Os dados evidenciam vários pontos críticos, que indicam áreas nas quais as expectativas formativas não foram atingidas:

– Planejamento de pesquisa dentro do marco ético e legal: um dos pontos mais críticos, com 68,78% dos respondentes sem atingir as expectativas. Isso reflete a necessidade de maior capacitação em pesquisa e conhecimento das regulamentações éticas.
– Avaliação de indicadores de estrutura, processo e resultado do centro de DP: com 59,12% de respostas críticas, os nefrologistas demonstram dificuldades em avaliar indicadores, habilidade fundamental para o gerenciamento eficiente dos centros de DP.
– Execução das trocas de diálise peritoneal ambulatorial contínua (DPAC) (31,5%) e conexões com cicladora (59,4%): há discrepâncias importantes, pois 31,5% dos respondentes não sabem realizar uma troca manual de DPAC, e 59,4% não têm experiência ou conhecimento em realizar conexão com cicladora. São aspectos práticos críticos que impactam diretamente a capacidade do profissional de prestar um cuidado adequado.
– Interação com a equipe multiprofissional: 32,04% relatam deficiências na interação com outros profissionais, sugerindo a necessidade de melhorar o trabalho em equipe e a coordenação no ambiente clínico.
– Participação na gestão de um centro de DP (55,52%) e análise crítica de indicadores (58,56%): mais da metade dos respondentes não se sente preparada para gerir adequadamente centros de DP ou realizar a análise crítica dos indicadores de desempenho.

**Tabela 6:** Resultados da pesquisa com nefrologistas latino-americanos sobre aprendizado

Pontos críticos	Nº de respondentes	Proporção crítica (%) (*não atendem às expectativas*)	Nº de casos (frequência)
Planejamento de pesquisa dentro do marco ético e legal	362	68,78%	249
Avaliação de indicadores de estrutura, processo e resultado do centro de DP	362	59,12%	214
Análise crítica de indicadores	362	58,56%	212
Interação com a equipe multiprofissional de cuidado	362	32,04%	116
Participação na gestão de um centro de DP	362	55,52%	201
Contribuição para a educação terapêutica de pacientes e cuidadores	362	34,25%	124
Realização de trocas de DPAC	362	31,50%	114
Realização de conexões com cicladora	362	59,40%	215

Abreviaturas – DP: Diálise Peritoneal; DPAC: Diálise Peritoneal Ambulatorial Contínua.

### Ponto de Discordância Principal

Um aspecto central que merece atenção adicional é a educação terapêutica dos pacientes. Apenas 124 respondentes (34,25%) afirmaram não atender às expectativas nesse quesito, o que poderia indicar que a maioria (238 respondentes) participa ativamente da educação dos pacientes. No entanto, esse dado é contraditório em relação a outros aspectos fundamentais, já que quase 60% dos respondentes não têm experiência no uso de cicladoras e 31,5% não sabem realizar uma troca manual de DPAC.

Isso cria uma discrepância preocupante: como é possível que a maioria dos profissionais afirme participar da educação terapêutica dos pacientes se não domina habilidades práticas essenciais, como o manuseio de cicladoras ou a troca manual? Educar adequadamente pacientes em DP requer domínio completo dessas técnicas. Essa inconsistência sugere uma possível superestimação das habilidades educacionais dos respondentes ou que os programas de educação de pacientes não abordam suficientemente essas áreas críticas.

### Políticas Públicas

A Tabela 7 ilustra os principais resultados obtidos na pesquisa realizada com 27 nefrologistas de 14 países latino-americanos, focada em aspectos-chave da diálise peritoneal. Esses resultados destacam percepções sobre a preferência pela DP como terapia de primeira linha, a existência (ou ausência) de regulamentações, barreiras econômicas e geográficas, bem como a necessidade de um marco legislativo robusto para promover a DP.

**Tabela 7:** Resultados da pesquisa sobre políticas públicas em diálise peritoneal

Ponto	Explicação
Preferência pela DP como primeira opção	91% dos respondentes concordam ou concordam fortemente que a DP é a melhor opção para iniciar a diálise em pacientes incidentes.
Legislação sobre DP	63% dos respondentes relatam que seus países possuem regulamentações para o desenvolvimento de programas de DP, mas apenas 50% afirmam que essas normas abrangem todos os aspectos necessários.
Consentimento informado	81% dos respondentes indicam que não existe lei que garanta o consentimento informado para que os pacientes conheçam todas as suas opções de tratamento (DP e hemodiálise).
Prioridade da DP como primeira opção	Apenas um país possui lei que estabelece a DP como tratamento inicial, mas não há mecanismos que assegurem sua aplicação efetiva.
Financiamento da DP	38% dos países não garantem financiamento integral para a DP e, em 25%, há necessidade de coparticipação financeira por parte dos pacientes.
Acessibilidade regional	69% dos respondentes relatam que a DP não está acessível em todas as regiões de seus países, sugerindo desafios logísticos e de infraestrutura.
Necessidade de uma lei que priorize a DP	87% dos respondentes acreditam que deveria existir uma lei que priorizasse e financiasse a DP como primeira opção de tratamento dialítico.
Barreiras à implementação da DP	As principais barreiras para a implementação da DP são a falta de educação e experiência, seguidas pela carência de financiamento e de políticas públicas adequadas.

Abreviatura – DP: Diálise Peritoneal.

Os achados revelam que, embora exista um amplo consenso na comunidade médica quanto aos benefícios da DP como terapia inicial para pacientes incidentes em diálise, há lacunas significativas na implementação e regulamentação dos programas de DP nos países participantes. A falta de regulamentações abrangentes, a ausência de leis de consentimento informado e a carência de mecanismos que garantam acesso equitativo à DP em todas as regiões são obstáculos que precisam ser enfrentados.

Além disso, a pesquisa evidencia que o financiamento insuficiente e a obrigatoriedade de coparticipações em alguns países limitam o acesso ao tratamento, especialmente para pacientes economicamente vulneráveis. Também se identificam barreiras educacionais e de experiência dos profissionais de saúde como fatores-chave que dificultam a expansão dos programas de DP.

Por fim, uma alta proporção de respondentes considera necessária a criação de leis que priorizem e financiem a DP como opção dialítica inicial, sugerindo que a implementação de um marco legislativo adequado é essencial para superar as barreiras atuais e melhorar o acesso à DP em toda a região.

### Acesso

A Tabela 8 apresenta os principais achados derivados da pesquisa sobre acesso sustentável e equitativo à DP na América Latina. Esses resultados refletem propostas para aprimorar a acessibilidade, a qualidade e a custo-efetividade da DP na região, baseadas em evidências científicas e nas experiências dos respondentes.

**Tabela 8:** Resultados da pesquisa com especialistas latino-americanos sobre acesso à diálise peritoneal

Achado / Proposta	Justificativa
Criar centros de DP em áreas urbanas e rurais para melhorar a acessibilidade	Aumenta o acesso à DP em regiões com infraestrutura limitada, garantindo a distribuição equitativa dos serviços.
Estabelecer unidades satélites sob supervisão de nefrologistas responsáveis	Assegura a qualidade do cuidado e a especialização mesmo em regiões com menor número de especialistas.
Utilizar sistemas de informação geográfica para identificar áreas de necessidade e planejar a expansão	Melhora a eficiência na alocação de recursos e no planejamento de novas unidades de DP com base na distribuição populacional.
Fornecer resultados científicos de custo-efetividade para embasar decisões de financiamento	Sustenta decisões baseadas em evidências que favorecem a DP como opção custo-efetiva e sustentável em comparação com a hemodiálise.
Criar modelos de financiamento eficientes que otimizem a equidade no acesso à terapia renal substitutiva	Melhora o acesso à DP como terapia mais custo-efetiva, especialmente em países com poucos recursos, reduzindo o impacto fiscal a longo prazo.
Estabelecer indicadores-chave para monitorar a qualidade e a eficiência dos programas de DP	Permite o monitoramento constante e eficaz dos programas, garantindo a manutenção de altos padrões de cuidado e sustentabilidade.
Desenvolver protocolos padronizados para DP de urgência, garantindo acesso imediato à terapia	Garante que os pacientes tenham acesso seguro e eficaz à DP em situações críticas, melhorando os desfechos clínicos.
Estabelecer unidades de curta permanência para pacientes que iniciam DP de urgência	Facilita a transição dos pacientes para a DP, permitindo o monitoramento intensivo nos primeiros dias de tratamento.
Implementar plataformas digitais e telemedicina para facilitar a educação contínua e o monitoramento remoto	Melhora a educação do paciente e o acesso ao acompanhamento remoto, otimizando o autocuidado e a adesão ao tratamento em regiões remotas.

Abreviatura – DP: Diálise Peritoneal.

São destacadas estratégias relacionadas à expansão de unidades de DP, à otimização do financiamento e ao uso de tecnologias para melhorar o planejamento e o monitoramento do tratamento.

Os resultados indicam que a expansão da DP é uma solução viável e custo-efetiva para reduzir as disparidades no acesso à terapia renal substitutiva, especialmente em áreas rurais e de difícil alcance. A criação de unidades satélites sob supervisão de nefrologistas e a implementação de sistemas de informação geográfica são estratégias-chave para garantir a distribuição equitativa dos serviços.

Além disso, deve-se aproveitar a evidência científica sobre o custo-benefício da DP em relação à hemodiálise para implementar modelos de financiamento mais eficientes, que assegurem um acesso equitativo e sustentável a longo prazo. O estabelecimento de indicadores-chave para monitorar a qualidade e a eficiência dos programas de DP permitirá o acompanhamento adequado dos resultados, garantindo que os pacientes recebam cuidados de alta qualidade.

Em conclusão, esses achados fornecem diretrizes claras para o fortalecimento das políticas e do planejamento estratégico em torno da DP, assegurando que essa modalidade terapêutica se torne uma ferramenta central para reduzir as disparidades no cuidado renal na América Latina.

## Discussão

A Declaração de Santo Domingo destaca a necessidade urgente de enfrentar as desigualdades na formação e no cuidado nefrológico e na diálise peritoneal (DP) na América Latina — um desafio compartilhado com regiões como a Europa, os Estados Unidos e a China. A variabilidade na infraestrutura e no acesso a recursos, somada às disparidades na formação, perpetua um cuidado desigual e subótimo.

Na Europa, ferramentas como os *Capabilities in Practice* (CiPs) e as *Generic Professional Capabilities* (GPCs), desenvolvidas pelo *General Medical Council* (GMC), foram implementadas para padronizar competências essenciais e assegurar qualidade na formação.

Contudo, sua aplicação enfrenta variações decorrentes das diferenças estruturais, semelhantes às observadas na América Latina^11,12^. Nos Estados Unidos, as barreiras à implementação de programas ideais de DP incluem limitações educacionais, regulatórias e financeiras, que afetam tanto pacientes quanto profissionais de saúde^13^.

Por outro lado, a China tem apresentado avanços expressivos na capacitação em DP, impulsionados pela Sociedade Chinesa de Nefrologia e pela criação de centros de excelência, que promovem padrões homogêneos e educação continuada. No entanto, persistem desigualdades entre áreas rurais e urbanas, afetando a formação em regiões menos desenvolvidas^14^.

Iniciativas como a proposta europeia para padronizar competências essenciais oferecem um modelo valioso para a América Latina, onde diferenças de infraestrutura e acesso se somam às disparidades formativas.

Assim como em outras regiões, a América Latina enfrenta grandes desafios na adoção da DP, marcados por disparidades regionais de uso, barreiras centradas no paciente, limitações dos centros de saúde e fragmentação dos sistemas de cuidado, além de uma abordagem excessivamente mecanicista que limita a integração de objetivos centrados no paciente, como propõe a iniciativa SONG.

Na Europa, as taxas de uso da DP apresentam fortes contrastes: enquanto os países escandinavos alcançam até 35% de adoção, na Grécia e na Romênia esse número fica abaixo de 5%. Além disso, apenas 12% dos pacientes com mais de 75 anos utilizam DP, apesar de seus benefícios comprovados^15^.

A iniciativa SONG, especialmente sua vertente SONG-PD, oferece um marco global para a padronização de desfechos em DP e para a priorização de resultados relevantes tanto para pacientes quanto para profissionais. Contudo, essa iniciativa não estabelece critérios de formação para as equipes de saúde.

Integrar esses padrões na região poderia melhorar a equidade e a eficácia dos programas de formação, garantindo que as competências profissionais estejam alinhadas aos desfechos clínicos mais relevantes^16^.

Na região, 59% dos médicos entrevistados não têm experiência com cicladoras, e 31,5% não sabem realizar trocas manuais de DP. Esses números refletem a falta de padronização nos programas de formação, um problema que poderia ser enfrentado com a adaptação de ferramentas como o *European Specialty Examination in Nephrology* (ESENeph), que, na Europa, garante a certificação de competências mínimas e facilita a mobilidade profissional^17^. Essas desigualdades dificultam a harmonização dos padrões na formação dos nefrologistas. Habilidades interpessoais e de comunicação, além de uma prática baseada nos sistemas de saúde, são essenciais para lidar com as complexidades do contexto assistencial latino-americano. A inclusão de rotações obrigatórias em atenção primária também poderia favorecer a prevenção e o manejo precoce das doenças renais^18^.

O impacto dessas desigualdades não se limita à formação, mas também afeta a qualidade do cuidado recebido pelos pacientes. No Reino Unido, o Relatório Francis destacou de que forma deficiências na formação e na supervisão podem comprometer a segurança do paciente^19^.

Ferramentas como o ESENeph poderiam ser adaptadas ao contexto latino-americano para garantir competências mínimas e facilitar a mobilidade profissional entre países^20^.

No campo das políticas públicas, a experiência de Hong Kong com a política *PD First* demonstra que priorizar a DP pode ser uma estratégia custo-efetiva e equitativa para o tratamento de pacientes com insuficiência renal crônica^21^. Na América Latina, embora 91% dos médicos entrevistados acreditem que a DP deva ser a terapia inicial preferencial, apenas El Salvador possui legislação específica que a prioriza. Barreiras financeiras, como os copagamentos, também limitam sua adoção em larga escala^22^.

A falta de acesso equitativo a terapias de alta qualidade, como a DP, reflete uma inequidade estrutural no cuidado médico, que afeta desproporcionalmente as populações vulneráveis. Integrar uma abordagem baseada em direitos humanos nas políticas de saúde renal é fundamental para garantir que todos os indivíduos, independentemente de sua localização ou condição socioeconômica, tenham acesso a essas terapias. Vanholder et al. destacam que as iniquidades em saúde renal não apenas limitam o acesso, mas também perpetuam desigualdades sociais já existentes^23^.

A criação de um marco regulatório com indicadores-chave para monitorar qualidade e acesso, apoiado por ministérios da saúde, sociedades científicas e organizações de pacientes, pode reduzir essas lacunas e assegurar que a DP seja acessível e financeiramente viável para todos.

O acesso desigual à DP também reflete um problema estrutural. Em áreas rurais, a falta de infraestrutura limita a disponibilidade de terapias domiciliares. Na Europa, o desenvolvimento de unidades satélites supervisionadas por nefrologistas especializados e o uso de telemedicina têm melhorado significativamente o acesso em regiões remotas^24^. Na América Latina, um exemplo promissor é o da Colômbia, com programas de impacto positivo em comunidades locais^25^.

Outro exemplo é o do Peru, onde se desenvolve um programa de visitas domiciliares a pacientes em DP, realizado por profissionais da atenção primária e financiado pelo *Fondo Intangible de Seguridad Social del Ministerio de Salud* ^26^.

### Ações Propostas

Diferentemente de países onde o acesso à DP é melhor estruturado e padronizado, a América Latina enfrenta problemas estruturais que precisam ser resolvidos antes de avançar para a padronização dos desfechos clínicos. Enquanto a iniciativa SONG-PD concentra-se em desfechos prioritários — como sobrevida técnica, redução de infecções e participação na vida diária —, na América Latina é necessário priorizar a organização dos processos fundamentais antes de implementar esses padrões.

O fortalecimento da formação profissional, a criação de políticas públicas robustas e a garantia de acesso equitativo são etapas preliminares essenciais para reduzir as desigualdades atuais e alcançar uma implementação mais eficiente da DP na região.

A Tabela 9 apresenta um conjunto de propostas estratégicas para melhorar o acesso, a capacitação e as políticas públicas relacionadas à DP na América Latina. Essas propostas, organizadas em torno dos macrodomínios de educação, políticas públicas e acesso, têm como objetivo fortalecer o sistema de saúde regional, promover a equidade e assegurar a formação contínua dos profissionais de saúde, bem como a educação dos pacientes.

**Tabela 9:** Propostas de ação da Declaração de Santo Domingo

Macrodimensão	Proposta	Descrição
Educação	Critérios mínimos de capacitação	Estabelecer critérios mínimos padronizados para o treinamento em DP, alinhados à SLANH-LACDD, abrangendo competências práticas como colocação de cateter e manejo de complicações, aplicáveis tanto a nefrologistas em formação quanto à educação continuada.
	Selo de qualidade para unidades de treinamento e rotação regional	Criar um selo de qualidade para certificar unidades de treinamento em DP na América Latina. Essas unidades devem contar com equipe qualificada, infraestrutura adequada e permitir rotações regionais em centros especializados, garantindo ampla exposição clínica e formação completa.
	Curso avançado on-line de DP	Desenvolver um curso on-line avançado de DP com fases de capacitação (inicial, básica, avançada e chefia de serviço), credenciado pela SLANH-LACDD e adaptado às necessidades locais. O curso incluirá módulos teóricos, simulações clínicas e um forte componente prático.
	Escola on-line para pacientes	Criar uma escola on-line para pacientes, coordenada pela SLANH-LACDD, para educação em manejo domiciliar da DP. A plataforma incluirá materiais multimídia e recursos acessíveis, fortalecendo a adesão ao tratamento e melhorando os desfechos clínicos.
	Desenvolvimento de materiais educacionais e uso de tecnologia avançada	Investir em materiais educacionais inovadores, como guias visuais, simulações interativas, videoaulas e no uso de inteligência artificial para personalizar o aprendizado. As plataformas tecnológicas devem incluir ferramentas de monitoramento remoto e lembretes automáticos para o manejo domiciliar.
	Capacitação em educação em saúde	Criar um programa de formação para educadores em saúde (médicos e enfermeiros) com base na andragogia, no construtivismo e em tecnologias educacionais. Os profissionais devem ser capacitados para elaborar programas significativos voltados a pacientes adultos em DP.
	Avaliação contínua do programa educacional	Implementar um sistema contínuo de avaliação baseado em rubricas para medir as competências adquiridas por pacientes e profissionais. As avaliações serão integradas à gestão da unidade de DP, melhorando a comunicação entre médicos e enfermeiros e acompanhando o progresso educacional do paciente.
	Desenvolvimento de programas de treinamento padronizados	Elaborar currículos padronizados para a formação de nefrologistas, enfermeiros e profissionais da atenção primária, adaptados às necessidades locais e com foco na DP como terapia domiciliar preferencial.
Políticas públicas	Acesso equitativo à terapia renal substitutiva	Garantir condições financeiras e operacionais em cada país da América Latina para reduzir as iniquidades no acesso à terapia renal substitutiva, por meio da implementação de um programa global de saúde que comece na atenção primária, com clínicas pré-diálise voltadas à decisão informada.
	Prioridade da DP como terapia domiciliar	Estabelecer uma política regional que favoreça a DP como terapia domiciliar, com regulamentações nacionais que eliminem a prática da diálise peritoneal intermitente (ambulatorial, quando o paciente faz a terapia somente na clínica de diálise). Essa política deve ser liderada pela SLANH-LACDD e pelo Organismo Andino de Salud – Convênio Hipólito Unanue.
	Registros nacionais de pacientes em terapias renais substitutivas	Criar ou fortalecer registros nacionais de pacientes em terapia renal substitutiva e em estágios 3 e 4 da doença renal crônica, melhorando o planejamento, o acompanhamento e a avaliação epidemiológica em cada país latino-americano.
	Avaliação econômica das terapias renais substitutivas	Realizar estudos de avaliação econômica das terapias renais substitutivas para otimizar recursos e subsidiar políticas baseadas em evidências, sob coordenação da SLANH-LACDD.
	Desenvolvimento de um marco legal para DP	Propor mudanças nas políticas de saúde de cada país, elaborando ou atualizando diretrizes clínicas que favoreçam a expansão da DP como terapia domiciliar, adaptando-as ao contexto latino-americano.
Acesso à DP	Formação de um comitê diretor	Estabelecer um comitê diretor com representantes dos ministérios da saúde, sociedades de nefrologia e organizações de pacientes, com o objetivo de definir papéis e responsabilidades na implementação do programa de DP e desenvolver um plano estratégico para sua expansão.
	Implementação de unidades satélites de DP e telemedicina	Promover a criação de unidades satélites de DP que atendam comunidades locais, incorporando tecnologias de telemedicina para facilitar o monitoramento remoto dos pacientes e as consultas especializadas, ampliando o acesso em áreas remotas.
	Expansão e avaliação do programa de DP	Expandir gradualmente o programa de DP nos países latino-americanos com base nos resultados de fases-piloto. Dados sobre acesso, desfechos clínicos e custos devem ser avaliados periodicamente para ajustes e implementação sustentável.
	Integração da DP aos sistemas universais de saúde	Trabalhar na incorporação da DP aos sistemas universais de saúde da região, garantindo cobertura e inclusão da DP como opção preferencial quando apropriado, fortalecendo os fluxos integrados de cuidado entre diferentes níveis assistenciais.
Integração de políticas e acesso	Grupo Internacional para Políticas e Acesso Renal (GIPAR)	Propõe-se a criação do GIPAR, uma equipe de apoio a políticas públicas composta por representantes da SLANH-LACDD, do Organismo Andino de Salud – Convênio Hipólito Unanue e das sociedades de nefrologia de cada país. O GIPAR coordenará ações com governos locais, elaborará propostas para melhorar o acesso às terapias renais substitutivas e garantirá a implementação de regulamentações que priorizem a DP como terapia domiciliar preferencial. Trabalhará na criação de um marco regulatório comum, baseado em boas práticas e adaptado à realidade de cada país, promovendo equidade e acesso universal à terapia renal substitutiva na região. Coordenadoras: Dra. Rosana Chaúd Covarrubias e Dra. Laura Cortés.

Abreviaturas – DP: Diálise Peritoneal; LACDD: Capítulo Latino-Americano de Diálise Domiciliar; SLANH: Sociedade Latino-Americana de Nefrologia e Hipertensão.

Cada proposta foi desenhada para enfrentar lacunas atuais no cuidado da doença renal crônica, desde a implementação de programas de capacitação até a criação de equipes internacionais dedicadas a impulsionar políticas públicas necessárias. A colaboração entre a SLANH-LACDD, o Organismo Andino de Salud – Convênio Hipólito Unanue, e as sociedades de nefrologia de cada país será fundamental para o êxito dessas iniciativas.

## Conclusões

A Declaração de Santo Domingo representa um marco importante na melhoria da diálise peritoneal na América Latina. No entanto, para que os compromissos aqui descritos se transformem em ações efetivas, é essencial a colaboração entre sociedades científicas, ministérios da saúde e universidades de cada país.

Somente por meio de um esforço conjunto será possível desenvolver políticas públicas adequadas, otimizar a formação dos profissionais de saúde e assegurar o acesso equitativo a tratamentos essenciais. Propõe-se, portanto, a criação de grupos de trabalho nacionais, nos quais esses atores-chave possam identificar barreiras locais e adaptar as estratégias da Declaração de Santo Domingo às necessidades específicas de cada região. Convidamos os líderes dessas instituições a participarem de encontros intersetoriais que permitam avançar rumo a um sistema de saúde mais inclusivo e eficiente para todos os pacientes com doença renal crônica na América Latina.

## Data Availability

Os conjuntos de dados gerados e/ou analisados durante o presente estudo estão disponíveis mediante solicitação razoável ao autor correspondente.
